# Coping Mechanisms and Resilience Strategies for Addressing Impostor Phenomenon in Healthcare Professionals

**DOI:** 10.7759/cureus.97367

**Published:** 2025-11-20

**Authors:** Sastry Chamarthi, Saketh Parsi, Usha Ravi, Rahul Kashyap, Venkata Sushma Chamarthi

**Affiliations:** 1 Department of Pediatrics, Clinica Sierra Vista Elm Community Health Center, Fresno, USA; 2 Department of Hospital Medicine, Ascension Seton Medical Center, Austin, USA; 3 Department of Pediatrics, Pediatric Associates, Tulare, USA; 4 Department of Medicine, Drexel University College of Medicine, Philadelphia, USA; 5 Department of Critical Care Medicine, Mayo Clinic, Rochester, USA; 6 Department of Research, WellSpan Health, York, USA; 7 Department of Pediatrics, Valley Children’s Healthcare, Fresno, USA

**Keywords:** coping mechanism, healthcare, healthcare worker well-being, impostor phenomenon, resilience strategies

## Abstract

The impostor phenomenon (IP) is a pervasive psychological challenge that affects healthcare professionals at all stages of training and practice. It is characterized by persistent self-doubt, feelings of inadequacy, and a fear of being exposed as incompetent, despite evidence of success. The high-stakes, hierarchical, and perfectionist culture of healthcare often amplifies these experiences. IP contributes to emotional distress, diminished professional performance, and potential effects on patient care, with frequent overlap with burnout, anxiety, and depression.

Addressing IP requires a multifaceted approach encompassing individual, peer, and organizational strategies. Individual interventions, such as cognitive restructuring, mindfulness practice, and self-efficacy training, help reframe maladaptive thought patterns and strengthen resilience. Peer-based strategies, including workshops, group discussions, and near-peer mentoring, reduce isolation and normalize shared experiences. At the organizational level, cultivating a culture of authenticity and psychological safety is essential. Structured mentorship, one-on-one coaching, role modeling, executive presence training, and the integration of wellness and resilience principles into the curriculum can foster sustained professional growth.

Although individual interventions may yield quicker relief, peer and organizational strategies provide essential reinforcement and long-term resilience. The most effective frameworks combine all three levels into comprehensive, continuous programs. Recognizing the broad implications of IP for both clinicians’ well-being and its impact on patient outcomes, healthcare systems should prioritize initiatives that promote coping and resilience.

## Introduction and background

The impostor phenomenon (IP), also known as impostor syndrome, is a psychological pattern in which individuals doubt their accomplishments and harbor persistent fears of being exposed as fraudulent, despite clear evidence of competence [1]. First described by Clance and Imes in 1978, IP was initially observed among high-achieving women but has since been recognized across diverse populations and professions [1]. In healthcare, IP manifests in distinctive ways, including a fear of inadequate patient care, persistent self-doubt despite clinical proficiency, and attributing success to external factors rather than one’s own skills. The phenomenon affects professionals across all career stages, from students and trainees to experienced clinicians, with prevalence rates of 44%-48%, exceeding those observed in other fields [2-5].

Healthcare environments provide particularly fertile conditions for the development of IP. High-stakes decision-making, rigid hierarchical structures, cultural expectations of perfectionism, and frequent role transitions inherent to training and clinical practice reinforce feelings of inadequacy even among competent practitioners [3,4]. Given the strong associations between IP and burnout, depression, suicidal ideation, and reduced professional performance, identifying and implementing effective coping mechanisms and resilience strategies have become an urgent priority for healthcare institutions worldwide [3,5-7]. This review synthesizes evidence-based interventions for addressing IP in healthcare professionals, organized across individual (cognitive-behavioral and mindfulness approaches), peer (workshops and mentoring), and organizational (mentorship programs and cultural change) levels, with emphasis on practical implementation strategies and sustained resilience-building.

## Review

Prevalence and impact on healthcare professionals

Impostor phenomenon is widely recognized as a significant occupational and psychological concern in healthcare, affecting individuals across diverse disciplines and career stages. Understanding both the scope of IP and its multifaceted impact on clinicians, trainees, and healthcare systems is essential for developing targeted, evidence-based interventions that promote resilience and professional fulfillment.

Prevalence Across Healthcare Professions

A comprehensive 2025 meta-analysis by Salari et al., examining 30 studies involving 11,483 healthcare providers, reported a global prevalence of 62% among health service professionals [2]. This high burden represents a growing public health concern because it affects not only individual clinicians but also the overall healthcare workforce, contributing to burnout, attrition, and a reduced capacity to meet patient needs. When large segments of the workforce experience persistent self-doubt and psychological distress, the downstream consequences include diminished quality of care, increased risk of medical error, and threats to healthcare system sustainability [8].

Prevalence varies considerably across professional categories and career stages, with medical students consistently demonstrating the highest rates. Rosenthal et al. found that 87% of first-year medical students at a university reported high or very high impostor phenomenon scores, highlighting vulnerability at the earliest stages of medical education [3]. In Sweden, Kristoffersson et al. noted that among 457 medical students, they reported a prevalence of 58.4% using validated assessment tools, noting that resilience was significantly inversely correlated with impostor feelings [4].

Among practicing physicians, Shanafelt et al. surveyed 3,116 US physicians. They found that they were approximately 30% more likely to experience the impostor phenomenon than other US workers, with strong associations with burnout and adverse mental health outcomes [5]. Physicians aged 35-44 years reported the highest impostor scores of any age group, suggesting IP persistence throughout career progression. Across studies, higher prevalence has also been documented among women and underrepresented minorities in medicine, indicating intersectional vulnerabilities that may require tailored interventions [9].

Impact on Professional Performance and Well-Being

The consequences of impostor phenomenon extend well beyond self-perception, affecting mental health, professional performance, and the quality of patient care. Strong associations have been observed with depression, anxiety, burnout, and suicidal ideation, with risk increasing in proportion to the severity of impostor experiences [5,7]. Professional functioning may be impaired by decreased engagement in learning, difficulty processing feedback, and the diversion of cognitive energy toward impression management, the excessive effort to maintain a façade of competence or conceal uncertainty [7]. These behaviors can reduce openness to mentorship, hinder authentic communication, and restrict growth opportunities.

In clinical contexts, impression management may delay help-seeking, discourage collaboration, and foster reluctance to voice concerns about patient safety. A qualitative study by Chodoff et al. among internal medicine residents described how IP cultivated unrealistic self-expectations, self-blame for patient outcomes, decision-making paralysis, and silence in moments when speaking up could have improved care [6].

Individual and peer-focused interventions

Efforts to mitigate impostor phenomenon often begin at the individual and peer levels, where self-awareness, reflection, and mutual support play key roles. Targeted interventions in these domains aim to challenge maladaptive thought patterns, normalize shared experiences, and foster a sense of belonging among healthcare professionals navigating high-pressure environments.

Cognitive-Behavioral and Mindfulness Approaches

Cognitive-behavioral therapy (CBT) is the most extensively studied individual intervention for the impostor phenomenon in healthcare settings. CBT techniques emphasize cognitive restructuring, helping professionals identify and challenge maladaptive beliefs such as “I don’t deserve this position” or “I just got lucky.” Studies report reductions in Clance Impostor Phenomenon Scale (CIPS) scores following structured cognitive-behavioral exercises that target attributional biases, although high-quality randomized trials in healthcare populations remain limited [7]. Attributional reframing methods, including the downward-arrow technique, help the participants uncover the core beliefs underlying impostor feelings through guided questioning of automatic negative thoughts [10].

Mindfulness-based stress reduction (MBSR) programs also show promising results in healthcare trainees and professionals. Participation in MBSR interventions has been associated with decreased distress, improved emotional regulation, and greater recognition of impostor-related thought patterns [11]. In one program, more than 80% of healthcare workers reported increased confidence in using mindfulness strategies to manage self-doubt [12]. Regular mindfulness practices, such as brief meditation, breathing awareness, or reflective journaling, appear to modulate stress reactivity and enhance self-compassion, promoting sustained psychological resilience. These interventions are particularly valuable for their accessibility, low cost, and ease of integration into medical education and clinical settings.

Self-Efficacy, Resilience, and Peer Support Strategies

Self-efficacy training directly targets the competency doubts central to the impostor phenomenon. Evidence indicates a strong inverse relationship between self-efficacy and impostor scores in healthcare professionals [13]. Structured programs that employ the SMART framework, Specific, Measurable, Achievable, Relevant, and Time-bound, support clinicians in building confidence through incremental mastery and progress tracking. Longitudinal studies suggest that fostering resilience and self-efficacy early in medical education reduces the persistence of impostor feelings later in professional life [14]. Professional development opportunities, such as supervisory responsibilities and structured feedback mechanisms, have similarly been linked to decreased impostor symptoms among surgeons [15].

Peer-based interventions complement individual approaches by reducing isolation and normalizing shared experiences. Workshops that combine didactic sessions, small-group discussions, and reflective exercises consistently yield high satisfaction, with participants reporting the improved recognition of impostor tendencies and the acquisition of effective coping strategies [16]. Near-peer mentoring (a model where a slightly more experienced individual mentors someone less skilled) and structured coaching models enhance collective support when mentor-mentee pairings are intentional and mentors receive basic facilitation training [17,18]. Recently, online peer-coaching platforms have emerged as scalable and accessible formats that preserve the interpersonal benefits of traditional peer groups while expanding their reach [18]. Together, these strategies highlight the importance of developing both personal coping skills and collegial support networks to enhance resilience among healthcare professionals.

Organizational and system-level interventions

While individual and peer-focused strategies address personal and interpersonal dimensions of impostor phenomenon, sustainable change requires institutional commitment. Organizational interventions aim to reshape workplace culture, policies, and leadership practices to foster psychological safety, normalize vulnerability, and integrate resilience-building into the fabric of healthcare systems.

Mentorship and Institutional Support

Mentorship programs represent a cornerstone organizational intervention for addressing the impostor phenomenon. Structured faculty-student mentorship, characterized by consistent supervision, psychologically safe feedback environments, and explicit acknowledgment of achievements, has been shown to improve professional self-perception across various healthcare settings [17,19]. Rosenthal et al. observed that supportive feedback, collaborative learning, and engaged faculty mentoring were particularly protective for medical students exhibiting impostor tendencies [3]. Effective mentorship models incorporate mentor training focused on recognizing impostor traits, normalizing vulnerability, and fostering open dialogue. When combined with peer-mentoring networks, these programs provide a continuous framework for belonging, professional growth, and longitudinal support throughout the training and practice period.

Early One-on-One Coaching

Imposter syndrome is systemic in nature and requires a multifaceted approach through educational programs, targeted interventions, and human resource management policies. Two primary intervention modalities focus on open discussion and counseling. Coaching, in particular, has consistently been shown to be effective, along with peer-exchange and support groups. These interventions not only enhance awareness and openness but also promote the practical application of coping strategies [20]. Several structured coaching programs have been developed to help physicians address the IP and build professional confidence. Such programs may be delivered through in-person sessions, phone consultations, or group meetings. Additionally, the online physician coaching program Better Together offers a supportive and personalized experience designed to help physicians overcome self-doubt while fostering both personal and professional growth [21].

Educating supervisors about the IP is critical to ensuring the success and well-being of trainees in healthcare settings. IP thrives in environments of silence and comparison; therefore, institutions can counter it by cultivating psychological safety, mentorship, and meaningful recognition. Through one-on-one coaching, leaders can help individuals identify their strengths, reframe negative self-talk, and build resilience [22,23]. Over time, such intentional structures can transform workplace culture, from one marked by quiet self-doubt to one defined by collective confidence and a sense of belonging, ultimately leading to the development of competent and confident healthcare professionals [10].

Training for Executive Presence

Executive presence, in the context of clinicians, refers to the ability to inspire confidence, demonstrate credibility, and communicate with clarity and empathy, particularly in complex, high-stakes healthcare environments [24]. In essence, executive presence integrates clinical competence with leadership poise, enabling clinicians to influence, guide, and reassure others, from the bedside to the boardroom.

Executive presence training can play a pivotal role in mitigating IP by fostering confidence, clarity, and authenticity in professional interactions. Through the mastery of communication skills, body language awareness, and emotional intelligence, clinicians learn to project credibility and self-assurance even in demanding situations [25]. This outward demonstration of composure reinforces internal confidence, helping individuals recognize their own competence and professional value.

Moreover, executive presence emphasizes purpose-driven leadership, aligning personal values with institutional goals. As clinicians cultivate the ability to lead with authenticity and influence, they begin to replace self-doubt with self-awareness [26]. This transformation nurtures psychological safety, strengthens peer trust, and fosters a workplace culture where clinicians feel empowered to contribute fully and fearlessly.

Aiding With Artificial Intelligence (AI)

In the modern era, the use of artificial intelligence (AI) in providing student feedback enables objective, near-real-time evaluation while allowing learners to reflect on their performance and progress. Innovations such as teacher-bots functioning as virtual teaching assistants can deliver instructional content, offer personalized feedback, and monitor student development. This efficient “fortifying feedback” loop not only enhances learning outcomes but also helps mitigate IP [27]. Additionally, several AI-driven platforms, such as Woebot and Wysa, extend their capabilities to coaching and mental health support, promoting self-awareness and emotional resilience among learners [28].

Cultural, Environmental, and Curricular Change

Institutional culture has a strong influence on the persistence and expression of the impostor phenomenon. Environments that perpetuate perfectionism and punitive norms can exacerbate self-doubt, whereas cultures emphasizing authenticity, vulnerability, and a growth mindset cultivate resilience. Mayo Clinic’s initiatives, such as storytelling forums where senior physicians share personal challenges, “failure resumes” presented during departmental meetings, and Colleagues Meeting to Promote and Sustain Satisfaction (COMPASS) groups, have been associated with improved collegiality, decreased stigma, and enhanced well-being among physicians [5]. These examples demonstrate the impact of systemic cultural reforms in promoting psychological safety and professional authenticity.

Medical education offers another critical avenue for preventive action. Competency-based curricula, small-group mentorship structures, and wellness integration mitigate competitive pressures and create safe spaces for reflection. Reflective writing exercises, resilience-building workshops, and wellness modules embedded throughout medical training have demonstrated effectiveness in reducing impostor tendencies during key stages of identity formation. When institutions integrate well-being and resilience into the formal curriculum, they signal a shift toward organizational accountability, viewing impostor phenomenon not as an individual deficit but as a shared cultural and systemic challenge.

Effectiveness, Duration, and Assessment

Comparative evidence suggests that multimodal interventions are more effective than isolated approaches. Zanchetta et al. found that individualized coaching was more effective than group training in reducing impostor phenomenon scores and the fear of negative evaluation, with benefits sustained at five-week follow-up [29]. However, group-based formats confer complementary advantages, including the normalization of experiences and the peer reinforcement of coping skills. The most effective models integrate individualized coaching, mentorship, and peer support within an institutionally endorsed framework that prioritizes well-being and professional development.

Sustained outcomes depend on the duration of the intervention and the reinforcement. Multi-session, longitudinal programs yield stronger and longer-lasting effects than single-session workshops [30]. The emergence of online coaching and peer-support platforms has expanded access and scalability, with recent systematic reviews documenting significant reductions in impostor-related symptoms among healthcare trainees participating in digital interventions [18]. Standardized measurement using validated tools, such as the Clance Impostor Phenomenon Scale (CIPS), allows for consistent outcome tracking [30]. Nonetheless, reliance on self-reported data highlights the need for objective evaluation metrics and longitudinal follow-up to assess actual behavioral and organizational impact.

**Figure 1:**
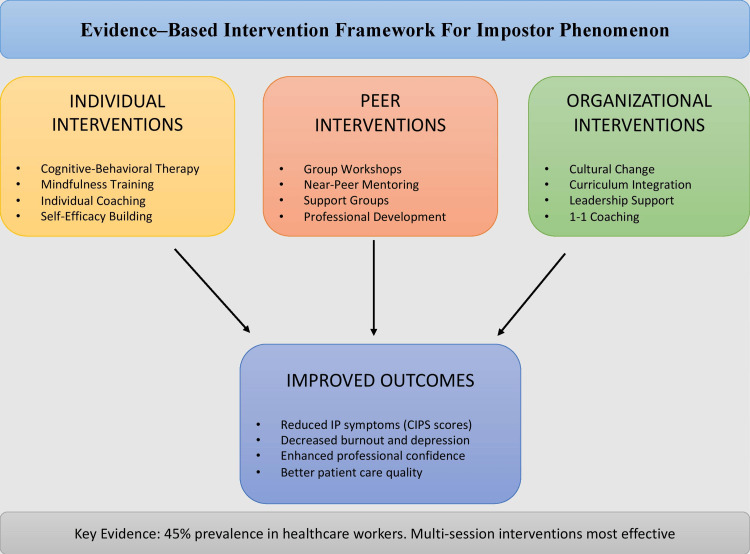
Evidence-based intervention framework for impostor phenomenon This framework illustrates three levels of interventions: individual (cognitive-behavioral therapy, mindfulness training, individual coaching, and self-efficacy building), peer (group workshops, near-peer mentoring, support groups, and professional development), and organizational (mentorship programs, cultural change, curriculum integration, and leadership support). Collectively, these approaches contribute to improved outcomes, including reduced impostor phenomenon (IP) symptoms, decreased burnout and depression, enhanced professional confidence, and improved patient care quality. Key evidence highlights a 62% prevalence of impostor phenomenon among healthcare workers, with multi-session, combined interventions demonstrating the most sustained effectiveness [3,7,9,10,12,16-18,20,29]. Figure created by author VSC CIPS: Clance Impostor Phenomenon Scale

An evidence-based framework summarizing key intervention strategies is presented in Figure 1.

To provide a concise overview of current approaches, all identified individual, peer, group, and organizational interventions for addressing impostor phenomenon in healthcare professionals are summarized in Table 1.

**Table 1:** Evidence-based interventions for impostor phenomenon in healthcare professionals This table summarizes validated individual, peer, group, and organizational interventions implemented in healthcare settings to mitigate impostor phenomenon. It outlines the implementation level, format or duration, target population, and key reported outcomes, with supporting references. Table created by author VSC CIPS, Clance Impostor Phenomenon Scale; IP, impostor phenomenon; COMPASS, Colleagues Meeting to Promote and Sustain Satisfaction

Intervention (with references)	Implementation level	Format/duration	Target population	Key outcomes
Cognitive-behavioral therapy [7]	Individual	Multi-session programs	Healthcare professionals	↓ CIPS scores; improved attribution patterns
Mindfulness-based stress reduction (MBSR) [12]	Individual	8-week course	Medical students and residents	↑ Confidence using mindfulness; ↓ distress
Individual/online coaching [18,20,29]	Individual/group	4-6 sessions (variable)	Physicians and trainees	↓ IP scores; improved accessibility
Reflective writing and attribution reframing [7,9]	Individual/group	Guided and multi-session	Medical trainees	↑ Self-awareness; improved recognition of competence
Interactive workshops [16]	Group	60-90 minutes and single or multi-session	Interprofessional teams	↑ IP awareness; high satisfaction
Peer or near-peer mentoring [3,10]	Peer	Structured and ongoing	Students and residents	↑ Support; ↓ anxiety; ↑ academic performance
Faculty-student mentorship [3,17]	Organizational	Ongoing supervision	Medical students	↑ Self-perception; ↓ impostor feelings
Curriculum/wellness integration [10]	Organizational	Embedded throughout training	Medical students	↓ Competitive pressure; ↑ collaboration
Cultural and institutional programs (e.g., COMPASS, storytelling, and failure resumes) [5]	Organizational	Long-term and system-wide	All clinicians	↑ Collegiality; ↓ perfectionism culture

Limitations and research gaps

Current research on interventions for impostor phenomenon in healthcare professionals remains constrained by several methodological limitations. Most studies rely on short-term, self-reported outcomes, with few evaluating durability beyond several weeks. Randomized controlled trials and well-defined control groups are limited, and many samples are dominated by female participants, reducing generalizability across the broader healthcare workforce. Specialty-specific analyses are also scarce, limiting insights into discipline-based variations. The lack of longitudinal follow-up further obscures whether interventions lead to sustained behavioral or cultural change. Additionally, few investigations examine organizational or contextual factors that influence program adoption and sustainability, leaving significant gaps in guidance for system-level implementation.

Clinical implications and future directions

Healthcare organizations may adopt comprehensive, multi-level strategies that address the individual, interpersonal, and institutional determinants of the impostor phenomenon. Core components include structured screening using validated tools, cognitive-behavioral and mindfulness-based interventions, peer workshops, mentoring and coaching programs, and cultural initiatives that promote psychological safety and executive presence. Effective implementation depends on visible leadership commitment, integration with existing wellness and professional development programs, tailoring to workforce diversity, and the allocation of sustainable resources with ongoing evaluation of outcomes. Future research should prioritize larger, multi-site randomized controlled trials across diverse healthcare contexts, as well as longitudinal studies that follow the participants from training through professional practice. Comparative-effectiveness research is needed to identify the most impactful intervention modalities, while specialty-specific analyses can clarify unique vulnerability profiles. Additional priorities include developing standardized protocols, assessing cost-effectiveness, leveraging digital-health platforms for scalable delivery, and examining how organizational culture influences program success. Understanding the experiences of underrepresented groups in medicine is essential, as they may face disproportionate exposure to the impostor phenomenon and distinct barriers to support.

## Conclusions

The impostor phenomenon represents a pervasive and consequential challenge across the healthcare professions, influencing clinicians’ well-being, workforce stability, and patient care quality. Evidence-informed interventions show meaningful promise, yet their long-term effectiveness depends on sustained strategies that integrate cognitive reframing, mindfulness, mentorship, and peer support within a supportive organizational culture. Multi-session, structured approaches yield the most substantial evidence to date. However, critical knowledge gaps persist regarding durability, comparative effectiveness, and scalability. Advancing rigorous, longitudinal research and embedding findings into institutional policy are essential next steps. By implementing coordinated, multi-level interventions and fostering environments that value authenticity and resilience, healthcare systems can meaningfully reduce the burden of impostor phenomenon and promote lasting professional fulfillment.
